# A qualitative study to elicit user requirements for lower limb wearable exoskeletons for gait rehabilitation in spinal cord injury

**DOI:** 10.1186/s12984-023-01264-y

**Published:** 2023-10-17

**Authors:** Diana Herrera-Valenzuela, Laura Díaz-Peña, Carolina Redondo-Galán, María José Arroyo, Lía Cascante-Gutiérrez, Ángel Gil-Agudo, Juan C. Moreno, Antonio J. del-Ama

**Affiliations:** 1 International Doctoral School, Rey Juan Carlos University, Madrid, Spain; 2 Biomechanics and Technical Aids Unit, National Hospital for Paraplegics, Toledo, Spain; 3 Biomedical Engineering Department, Superior Technical School of Telecommunications Engineering, Rey Juan Carlos University, Fuenlabrada, Madrid, Spain; 4 Physical Medicine and Rehabilitation Department, National Hospital for Paraplegics, Toledo, Spain; 5 Fundación del Lesionado Medular (Spinal Cord Injured Foundation), Madrid, Spain; 6 Unit of Neurorehabilitation, Biomechanics and Sensorimotor Function (HNP-SESCAM), Associated Unit of R&D&I to the CSIC, Toledo, Spain; 7 Neural Rehabilitation Group, Cajal Institute, CSIC–Spanish National Research Council, Madrid, Spain; 8 School of Science and Technology, Department of Applied Mathematics, Materials Science and Engineering and Electronic Technology, Rey Juan Carlos University, Móstoles, Madrid, Spain

**Keywords:** Usability/acceptance measurement and research, Experience, Qualitative methods, Usability testing and evaluation, Physical disabilities

## Abstract

**Objective:**

We aim to determine a comprehensive set of requirements, perceptions, and expectations that people with spinal cord injury (SCI) and the clinicians in charge of their rehabilitation have regarding the use of wearable robots (WR) for gait rehabilitation.

**Background:**

There are concerns due to the limited user acceptance of WR for gait rehabilitation. Developers need to emphasize understanding the needs and constraints of all stakeholders involved, including the real-life dynamics of rehabilitation centers.

**Methods:**

15 people with SCI, 9 without experience with WR and 6 with experience with these technologies, and 10 clinicians from 3 rehabilitation centers in Spain were interviewed. A directed content analysis approach was used.

**Results:**

78 codes grouped into 9 categories (physical results, usability, psychology-related codes, technical characteristics, activities, acquisition issues, context of use, development of the technologies and clinical rehabilitation context) were expressed by at least 20% of the users interviewed, of whom 16 were not found in the literature. The agreement percentage between each group and subgroup included in the study, calculated as the number of codes that more than 20% of both groups expressed, divided over the total amount of codes any of those two groups agreed on (≥ 20%), showed limited agreement between patients and clinicians (50.00%) and between both types of patients (55.77%). The limited accessibility and availability of lower limb exoskeletons for gait rehabilitation arose in most of the interviews.

**Conclusions:**

The limited agreement percentage between patients and clinicians indicates that including both types of users in the design process of these technologies is important, given that their requirements are complementary. Engaging users with prior technology experience is recommended, as they often exhibit strong internal consensus and articulate well-defined requirements. This study adds up the knowledge available in the literature and the new codes found in our data, which enlighten important aspects that ought to be addressed in the field to develop technologies that respond to users’ needs, are usable and feasible to implement in their intended contexts.

**Application:**

The set of criteria summarized in our study will be useful to guide the design, development, and evaluation of WR for gait rehabilitation to meet user’s needs and allow them to be implemented in their intended context of use.

**Supplementary Information:**

The online version contains supplementary material available at 10.1186/s12984-023-01264-y.

## Introduction

During the last 30 years, we have witnessed an increase in the development and testing of robotic wearable (WR) exoskeletons for walking rehabilitation following spinal cord injury (SCI). The intended effect of these devices is to induce neuroplastic changes through intensive walking training [1], while also providing task-related visual and functional feedback [2]. However, clinical evidence is still limited and nonconclusive [3], thus, the scientific community is questioning the design and application principles of WR, as well as pointing towards the actual understanding of how to tune WR control parameters depending on the patient’s characteristics and therapeutic goals [4]. Besides, WR have to provide a richer walking experience, allowing independent ambulation while maintaining postural stability [5].

In parallel, the scientific community is also becoming interested in the limitations related to users’ acceptance of WR and their interactions. Qualitative research allows exploring this phenomenon from the user’s point of view. Researchers have studied the perception of both patients and clinicians about the technologies after one session with a WR through face-to-face interviews [6, 7], using online surveys with only patients [8–10] or clinicians [8, 10], or to assess the number of developers that include users through the development of the technologies [11]. Longer studies have also been performed, where the authors evaluate patients’ [9, 12–20] or clinicians’ [19, 21] perception after receiving training with a WR, some of them with evolutive follow up throughout the study [9, 14, 16, 19]. Overall, the authors highlight the limited evaluation of user satisfaction with WR [22], the need to improve the usability of the devices [23], and the lack of reliable and valid instruments to assess the devices from the user’s perspective [24]. As a consequence, authors highlight the urge to involve people with neurological injuries in the design of WR, to develop devices that meet their needs [25–27], because users may only accept a technology if it is useful for their own purposes [28].

Therefore, the limited clinical evidence regarding WR for gait rehabilitation, their lack of customization, and the constraints in user acceptance, arise doubts as to whether it is worth investing in these pricey technologies [23], since there is no clear sustainable economic model to effectively deploy them. Therefore, developers need to study the bigger picture regarding WR for gait rehabilitation, emphasizing the understanding of the needs and constraints of all stakeholders involved: subjects with neurological injuries as primary users, clinicians and caregivers as secondary users, and the real-life dynamics of rehabilitation centers. In this study, we aim to determine a comprehensive set of requirements, perceptions, and expectations that people with spinal cord injury and the clinicians in charge of their rehabilitation have regarding the use of WR for walking rehabilitation, by using a directed content analysis approach. This qualitative research methodology enables requirements elicitation from the users’ perspective, encompassing both the knowledge available in the literature, and allowing new criteria to emerge from the new data collected through interviews. We hope the complete set of criteria summarized in our study will be useful to guide the design, development and evaluation of WR for gait rehabilitation to make sure the efforts invested in the field lead to technologies that respond to the needs and expectations of their primary and secondary end users and are feasible to implement in their intended context of use.

## Methods

### Study design

A qualitative study using directed content analysis was conducted [29], following the Standards for Reporting Qualitative Research [30] and Consolidated Criteria for Reporting Qualitative Research [31]. This design was chosen to state a set of requirements for the design and development of these technologies, taking as a starting point the requirements found in the literature [32, 33]. Criteria proposed in [34, 35] were followed to establish trustworthiness and credibility in line with similar qualitative research studies [36, 37]. The procedures used regarding data credibility, transferability, dependability, and confirmability are shown in Table 1 [38].

**Table 1:** Criteria and strategies used to establish trustworthiness

Criteria	Strategies used
Credibility	Investigator triangulation: the analysis of each interview was checked by two researchers. Additionally, both authors discussed all the analyses to reach consensus about the differences in coding and identified categories together Participant triangulation: the study included participants with different: degrees of experience with lower limb wearable exoskeletons, backgrounds, SCI classification, mobility impairment, ages, sex and related to different institutions. Therefore, multiple perspectives were acquired about a common topic: the requirements, expectations and needs of people with SCI for a wearable lower limb exoskeleton Triangulation of methods of data collection: semi structured interviews as well as researcher field notes were gathered Researcher reflexivity was reinforced by discussing researchers’ positionality in reference to the topic studied and the population included in the study, and by clarifying the rationale behind the study
Transferability	The methodology used in this study is described in-depth, including characteristics of researchers, participants, contexts and sampling strategies, as well as the procedures used for data collection and analysis
Dependability	Audit trail: the researchers kept record of all the steps taken during the process from the conception of the study to the reporting of the results. This register of the research path guarantees the study conform to the standards for qualitative research using content analysis
Confirmability	Triangulations of researchers, participants, and methods of data collection were performed Researcher reflexivity was reinforced by discussing researchers’ positionality in reference to the topic studied and the population included in the study, and by clarifying the rationale behind the study Relevant issues regarding the positioning of the researchers are: (a) the study is part of a larger project called TAILOR (RTI2018-097290-B-C31), aimed at developing “Personalized Robotic and Neuroprosthetic Modular Wearable Systems for Assistance of Impaired Walking”, (b) none of the researchers has a SCI, (c) none of the interviewers had ever developed a robotic technology or is in charge of developing the exoskeleton in TAILOR, and (d) the interviewers did not and will not provide any type of clinical assistance to the subjects recruited

### Literature survey

The initial codes and categories to implement the directed content analysis methodology were established based on a literature survey. An advanced search in the Scopus database was performed comprising the period until December 31st, 2020, using the query string “(exoskeleton) AND (user AND center* AND design) OR (perception) OR (experience) OR (perspective*)”, only research articles and reviews written in English were considered. Further selection was performed by reading the title and abstract, when necessary, to guarantee that the included articles assessed lower limb exoskeletons for gait rehabilitation in terms of the users’ perspective or experience.

### Context

The National Hospital for Paraplegics (HNP) is the main monographic public hospital for intensive rehabilitation of SCI in Spain. Institute Guttmann (IG) is the main private foundation for rehabilitation of neurological injuries in the region of Catalonia. Both have vast experience in cooperating in research projects devoted to the development and evaluation of rehabilitation technologies, including lower limb exoskeletons. Spinal Cord Injury Foundation (FLM) is a private neurorehabilitation center located in Madrid, Spain, that provides integral rehabilitation, including therapy with lower limb WR, for people with SCI after their discharge from rehabilitation hospitals.

### Participants

Both people with SCI and clinicians were separately considered as end-users, as they might have different perspectives and requirements. In addition, and to better understand the impact of the technology on patient's expectancies, we split the SCI group within patients with and without previous experience with WR (e-SCI and n-SCI respectively). We aimed at assessing different perspectives: feedback about actual technologies and requirements that arose from experience (e-SCI and clinicians), expectations and unbiased requirements for the technologies (n-SCI), and expert advice on the requirements for the technologies to be effective as a gait rehabilitation tool (clinicians). The common exclusion criteria were inability to communicate in Spanish, inability to use to use crutches or a walker to walk with the WR, difficulties in comprehension and communication, and refusal to participate in the study. This research complied with the Declaration of Helsinki and was approved by the Institutional Review Board at the Ethics Committee of the Hospital Complex of Toledo, Spain (CEIC-CHTO, no. 2541 17/02/2021). Informed consent was obtained from each participant.

### Participant recruitment

The sample size was determined following the estimates presented in [39], where it is reported that 15 to 20 interviews are required for content analysis to reach data saturation. We confirmed data saturation within each group after completing the sample. Besides, our sample size is consistent with previous similar studies using semi-structured interviews (3 to 17 subjects, median: 10) [6, 13, 17, 19, 21, 40–43]. SCI participants were recruited between March 30, 2021, and March 11, 2022 through criterion and convenience sampling techniques [39]: 9 from HNP (n-SCI) and 6 from FLM (e-SCI), aged from 20 to 65 years (see Table 2). No participants withdrew from the study.

**Table 2:** Demographic and clinical characteristics of the subjects with SCI recruited

Characteristic		Type		HNP (n = 9)							FLM (n = 6)					
Sex		Female		2 (22.2%)							2 (33.3%)					
Age		20–30		1 (11.1%)							1 (16.7%)					
		31–40		1 (11.1%)							1 (16.7%)					
		41–50		3 (33.3%)							2 (33.3%)					
		51–60		3 (33.3%)							1 (16.7%)					
		> 60		1 (11.1%)							1 (16.7%)					
AIS		A		1 (11.1%)							3 (50.0%)					
		B		0 (0.0%)							1 (16.7%)					
		C		3 (33.3%)							2 (33.3%)					
		D		5 (55.6%)							0 (0.0%)					
Time since injury		Mean ± STD Range (min–max)		5.9 ± 3.0 months (2.5–11) months							17.1 ± 12.2 years (2.7–30) years					
Injury level		C1–C8		3 (33.3%)							1 (16.7%)					
		T1–T6		3 (33.3%)							0					
		T7–T12		2 (22.2%)							4 (66.7%)					
		L1–L5		1 (11.1%)							1 (16.7%)					
WISCI II level	Level No subjects		0		9	12	16	19	20	0		6	9	20		
			1		1	1	1	2	3	2		1	2	1		
Etiology		Trauma		6 (66.7%)							5 (83.3%)					
		Others (Spinal sugery or spinal cord affections)		3 (33.3%)							1 (16.7%)					
Interview time (min)		Mean ± STD		34.0 ± 24.4							22.5 ± 12.6					
		Total recorded time		306.4							134.8					
Maximum educational level completed		Primary (mandatory)		1 (11.1%)							0					
		Secondary (mandatory)		2 (22.2%)							2 (33.3%)					
		High school (opt.)		1 (11.1%)							2 (33.3%)					
		Vocational training (FP)		1 (11.1%)							2 (33.3%)					
		University degree		3 (33.3%)							0					
		Postgraduate degree		1 (11.1%)							0					

Similarly, for the clinicians user group, physical medicine and rehabilitation physicians (PM&R) and physiotherapists (PT) involved with the rehabilitation of people with SCI and/or with experience in research with WR were recruited through convenience and snowball sampling techniques [39] between April 25th and May 19th, 2021: 6 from HNP and 4 from IG (see Table 3). No participants withdrew from the study.

**Table 3:** Demographic and professional characteristics of the clinicians recruited

Characteristic	Type	Total (n = 10)	HNP (n = 6)	IG (n = 4)
Sex	Female	5 (50.0%)	4 (66.7%)	1 (50.0%)
Profession	PM&R	5 (50.0%)	2 (33.3%)	3 (75.0%)
	Physiotherapist	5 (50.0%)	4 (66.7%)	1 (25.0%)
Age	Mean ± STD	41.8 ± 12.7	35.2 ± 10.6	51.8 ± 8.6
	Range (min–max)	(26 to 62 y.o.)	(26 to 56 y.o.)	(41 to 62 y.o.)
Years working with SCI people	Mean ± STD	12.2 ± 10.6	5.9 ± 5.2	21.5 ± 10.0
Self-perceived knowledge about lower limb exoskeletons	Mean ± STD (1 to 4 scale)	2.8 ± 0.6	2.5 ± 0.6	3.25 ± 0.5
Interview time (min)	Mean ± STD	21.2 ± 7.3	19.1 ± 8.8	24.3 ± 2.8
	Total recorded time	212.2	114.8	97.4

### Data collection

Individual, semi-structured interviews led by a theme-based interview guide with open-ended questions were used to obtain detailed descriptions of the themes previously identified in the literature (see Additional file 1: Annex 1) [39, 44]. The interviews were audio-recorded with written permission of the participants. When needed, follow-up questions to enhance the depth of the description of a specific topic were made. All the interviews were individual and conducted by one researcher in Spanish, they were scheduled according to participants availability. A total of 653.4 min were recorded, with an average of 26.1 ± 17.0 min per interview (see Tables 2 and 3).

### Data analysis

**Fig. 1:**
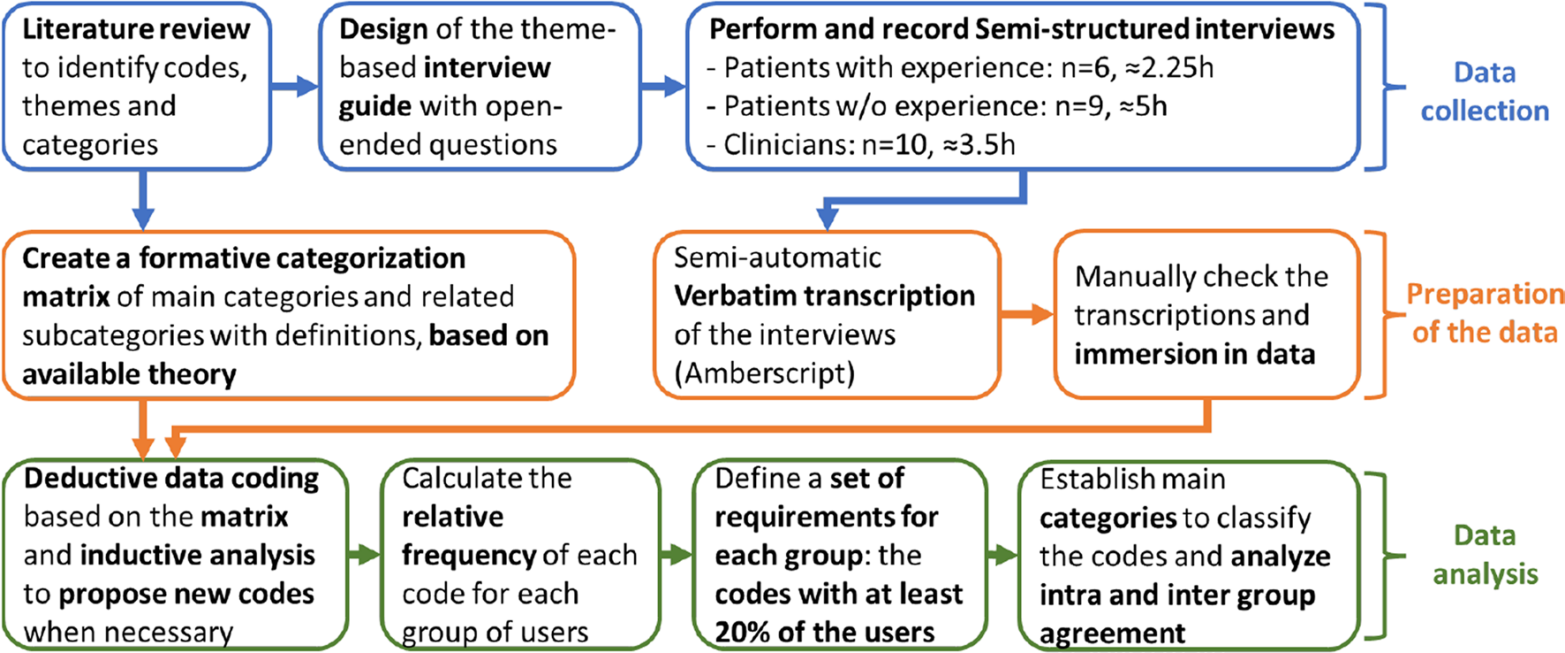
Details of the directive content analysis methodology implemented in the study

Verbatim transcriptions of all interviews were made using the semi-automated transcription software Amberscript (www.amberscript.com, Amsterdam, The Netherlands), these were reviewed and corrected manually. Two authors analyzed each transcription performing deductive content analysis, following the directive content analysis approach [45]. To this end the authors used a formative categorization matrix of the main categories and related subcategories, built based on the available literature [29, 46]. Afterwards, researchers performed an inductive analysis of the data based on the participants’ narratives to allow new codes and categories to emerge, thus extending and validating a conceptual framework [45]. Coding was conducted by both authors until a consensus was reached. The relative frequency of participants from each one of the three groups that referred to each code was calculated. The final list of requirements comprises only the codes with at least 20% of agreement within each group. Likewise, the intra and inter group agreement rate was calculated. Lastly, the authors identified categories and created a codebook. Data was organized and visualized using Microsoft Excel (www.microsoft.com/microsoft-365/excel, Redmond, WA, USA). The whole codification process is shown in Fig. 1.

## Results

### Literature survey

**Fig. 2:**
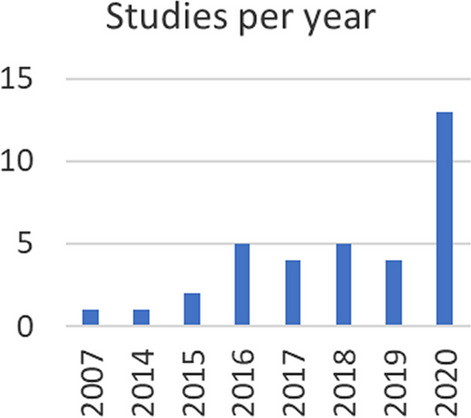
Number of articles published each year assessing lower limb exoskeletons for gait rehabilitation in terms of the user’s perspective or experience

The initial search yielded 53 results, of whom 32 studies were excluded after reading the title and abstract. The 21 articles included were analyzed in detail to compile the codes available in the literature regarding the design of lower limb exoskeletons for rehabilitation from a user-centered perspective [6, 8, 10, 12, 13, 15, 17, 21, 40–42, 47–56]. From the references of these articles, other 14 studies were identified as relevant and included in the analysis (see Fig. 2) [7, 9, 11, 14, 16, 18–20, 24, 25, 57–60].

The set of reviewed articles included diverse qualitative and quantitative methodologies to assess the perception or experience of users about lower limb exoskeletons. To create the categorization matrix, all the codes and themes that arose from qualitative methods such as content or thematic analysis, as well as the items of standardized questionnaires used in the studies were listed and grouped. In total, 98 codes were identified and grouped into 9 categories: physical results (21), usability (17), psychology related codes (15), technical characteristics (14), activities (12), acquisition issues (4), context of use (3), development of the technologies (5), and clinical rehabilitation context (7). The full list is available in Additional file 2: Annex 2. These requirements arose from the following stakeholders: patients, physiotherapists, occupational therapists, engineers, and salespersons.

### Participant recruitment and data collection

Our sample of primary users consisted of 15 adults with spinal cord injury (4 women, 26.67%), aged 45.5 ± 13.2 years (range 20 to 65 years), and with a median of 7.1 months since injury onset (min. 2.5 months, max. 30 years). All participants had a diagnosis of SCI with various degrees of impairment to walk, different etiologies of the injury (mostly traumatic, 73.3%), and a variety of injury classification (see Table 2). Subjects recruited had a wide variety of occupations and educational levels. Heterogeneity of the subjects was desirable to gather narratives from different perspectives.

As secondary users, 10 clinicians were recruited (5 women, 50.0%): five PM&R and five PT. Average age was 41.8 ± 12.7 y.o. (range 26 to 62 years), and had on average 12.2 ± 10.6 years of experience working with SCI (range 2 to 30 years) (see Table 3). Most of the subjects recruited had experience in clinical research of lower limb wearable robots for rehabilitation (n = 7, 70%), of whom three were also actively involved in clinical activity at the time of the study. The remaining three subjects had used the technologies in rehabilitation settings and were actively involved in clinical rehabilitation when interviewed. Experience and deep knowledge about lower limb exoskeletons for rehabilitation of spinal cord injury subjects was desirable to identify requirements that these devices must have to be a useful tool for gait rehabilitation.

### Codes and categories 

**Fig. 3:**
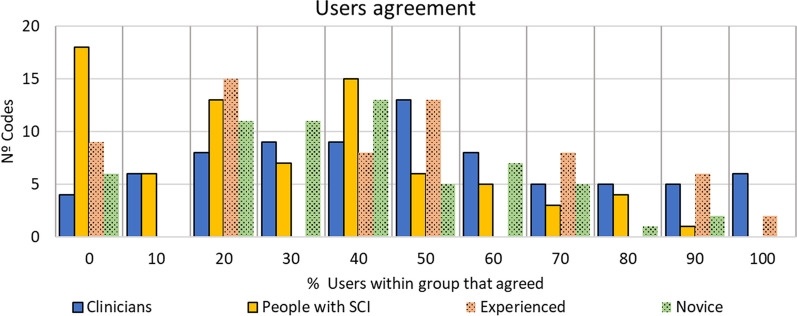
Intragroup agreement assessed as the number of codes (y-axis) that certain percentage of users of each group talked about (x-axis)

In total, 78 codes were retrieved from the interviews. Only the codes that were expressed by at least 20% of all the users interviewed were included. From these, 16 codes (20.25%) were not previously identified in the literature (see Table 4). In parallel, some codes available in the literature were merged during the analysis. All codes were classified in the previously stated categories: physical results (16), Technical characteristics (15), Usability (12), Psychology related codes (10), Activities (7), Development of the technologies (6), Clinical rehabilitation context (6), Context of use (4), and Acquisition issues (2). The narratives of the participants to describe each code were extracted directly from the interviews [29]. These are included in Additional file 2: Annex 2 with a detailed summary of the categories and the new codes retrieved in our study. Figure 3 shows the intragroup agreement rates.

**Table 4:** Number of codes that each group and subgroup of users talked about

Group	All users	Clinicians (n = 10)	People with SCI (n = 15)	e-SCI (n = 6)	n-SCI (n = 9)
Total nº of codes expressed	118	100	82	59	72
Nº codes agreed by ≥ 20% of the group	78 (66.10%)	82 (82.00%)	52 (63.41%)	38 (64.41%)	44 (61.11%)
New codes agreed by ≥ 20% of the group	16	11	10	8	8

We also assessed the agreement percentage between each group and subgroup included in the study, calculated as the number of codes that more than 20% of both groups expressed, divided over the total amount of codes any of those two groups agreed on (≥ 20%). This analysis showed that patients and clinicians agreed on 50.00% of the codes, n-SCI and e-SCI agreed on 55.77% of the codes, clinicians and e-SCI agreed on 45.83% of the codes and lastly, clinicians and n-SCI agreed on 45.45% of the codes.

**Fig. 4:**
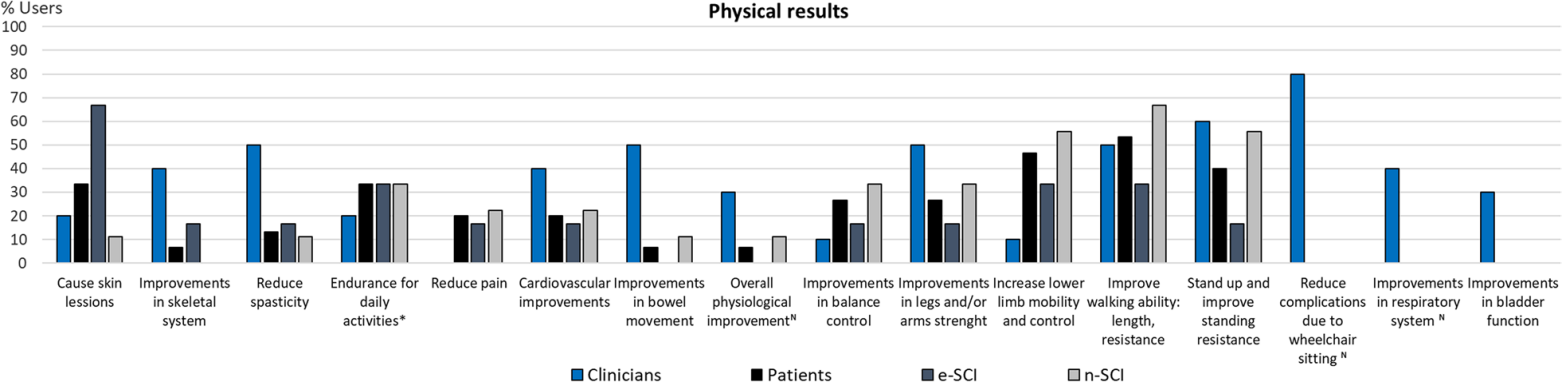
Codes of the category Physical results (n = 16). This is the category with more codes, most likely because the main goal of WR for gait rehabilitation is providing physical benefits. Firstly, we observe that the agreement of patients in these codes is low, especially in e-SCI. Clinicians have higher agreements and refer to more codes than patients, since physical benefits are the reason why they would use the technologies. Interestingly, this is the only category where n-SCI referred to more codes than e-SCI. Among patients, only e-SCI expressed the importance of having devices that do not cause skin abrasions, one of the most common adverse events related to the use of exoskeletons [23]. Users expect improvements not only related to walking and standing but also regarding other body systems that are benefited by walking, standing and in general, by avoiding long-lasting wheelchair sitting. One patient expressed that he did not expect the technologies to improve his endurance for daily activities, whereas more than 30% of all the patients did expect this. Three (3) new codes arose in this category: reduce complications due to wheelchair sitting, improvements in respiratory system, and overall physiological improvement

**Fig. 5:**
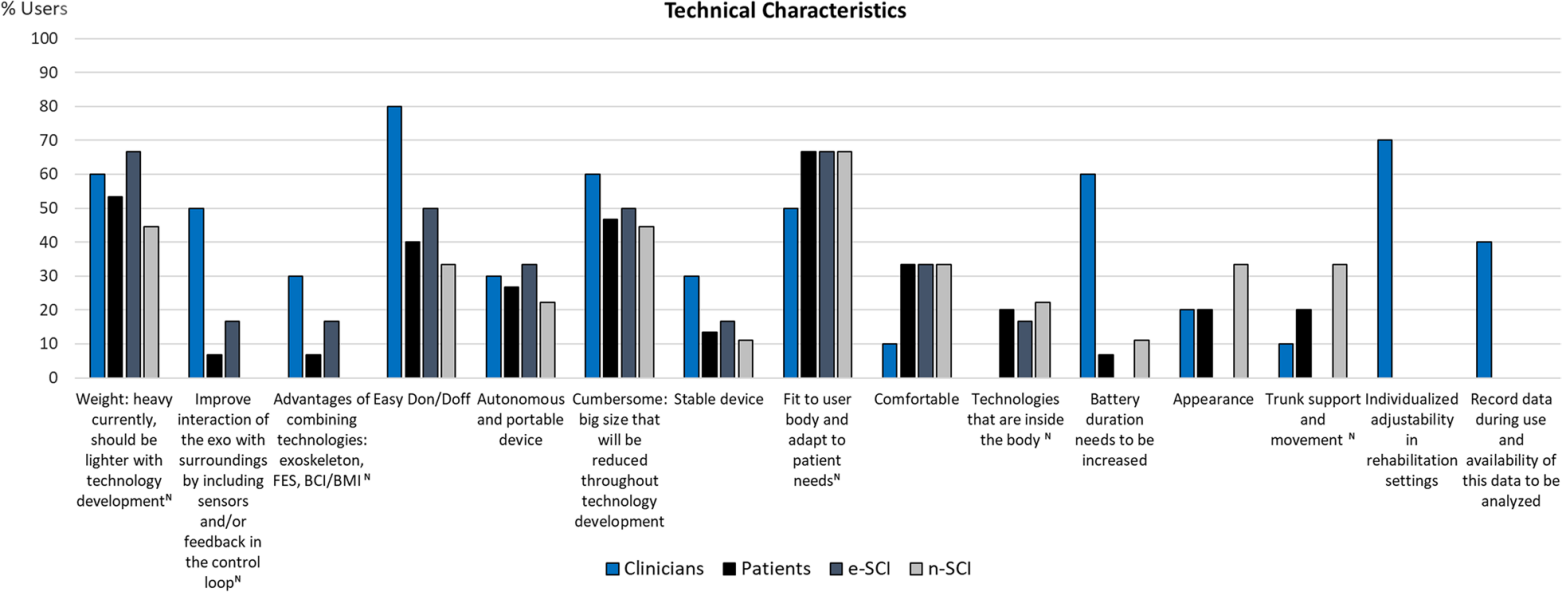
Codes of the category Technical characteristics (n = 15). This category comprises requirements that can translate directly into technical characteristics of the device and is the second largest one. Most of the codes within this category are expressed by at least 30% of the clinicians and two codes were only expressed by these users: (1) the possibility to adjust the device to each patient for rehabilitation and (2) to record and use the data gathered by the exoskeleton as feedback of the rehabilitation. Other codes with high agreement, expressed mostly by e-SCI and clinicians include: lowering the weight of the device and making it less cumbersome, easy donning and doffing (with the highest agreement among clinicians, given that this is essential to make the use of technologies viable during rehabilitation), and having a device that can be fitted to the body of each patient and able to adapt to the changing needs during the rehabilitation process. This is a new code and is the one with the highest agreement among all groups in this category. Additionally, mostly clinicians referred to the need of increasing the duration of the battery of the device and of improving the interaction of the devices with the surroundings by adding feedback in the control loop to allow the device to adapt. Almost one third of n-SCI considered important the device’s aesthetics, whereas no e-SCI talked about this. Interestingly, one code in this category is the mistaken expectation of some patients that exoskeletons will be embedded inside their bodies, an inconsistency with the definition of these devices showing that some patients expect long-lasting surgical aids or treatments for their rehabilitation instead of external tools for occasional use. Six (6) new codes emerged in this category, only two of them have not been addressed in this section: some users expect exoskeletons to provide trunk support and assist trunk movement, and some clinicians and e-SCI consider that combining exoskeletons with other technologies such as functional electrical stimulation (FES) or brain-computer interfaces (BCI) has advantages for users

**Fig. 6:**
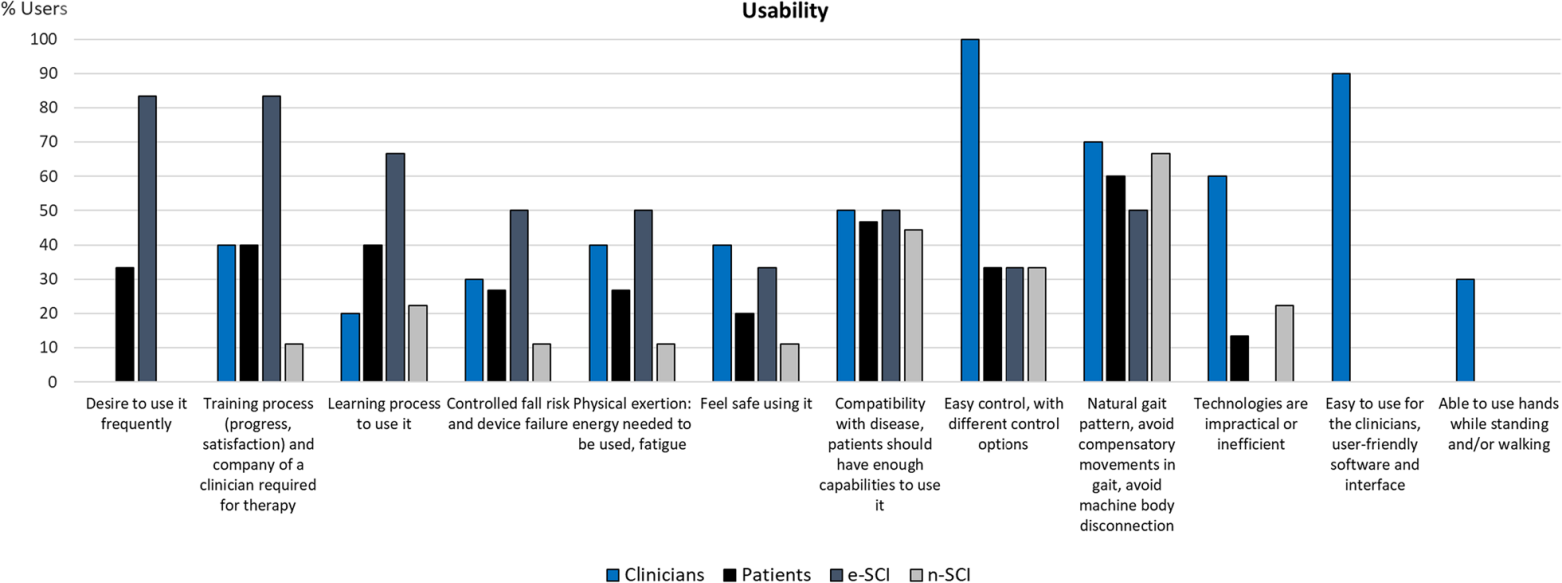
Codes of the category Usability (n = 12). Usability is not well defined in the field of WR [61], but in this category we grouped codes related to the interaction between the users and the technology that determine the outcome of its use. All but one e-SCI (83.33%) expressed their desire to use these devices more frequently, which is directly related to the limited accessibility that exoskeletons currently have. This was not mentioned by clinicians or n-SCI, most likely because the latter have not experienced this issue, since they have never used the technologies. Both e-SCI and clinicians agreed on the importance of the training process, which few n-SCI thought about during the interview. Likewise, patients and clinicians agreed on the relevance of having technologies that are compatible with all the clinical symptoms of the neurological injuries they are aimed for, and on having devices with gait patterns that avoid functional compensations, are natural, and allow the users to feel the connection with the machine. Some codes are related to the safety of the device: having the fall risk and device failure under control and providing a safety perception. The clinicians also highlighted the importance of the devices being easy to use by them and of allowing patients to use their hands while walking or standing. Regarding this last code, even though patients did not explicitly say it, various of the activities they expect to do with exoskeletons imply being able to use their hands freely. About the physical exertion caused by the devices, patients expressed that the energy needed to use the device at the beginning is very high but that after learning to use it, they expect to (n-SCI) and actually require (e-SCI) less effort to walk when compared to non-assisted walking. No new codes arose regarding usability

**Fig. 7:**
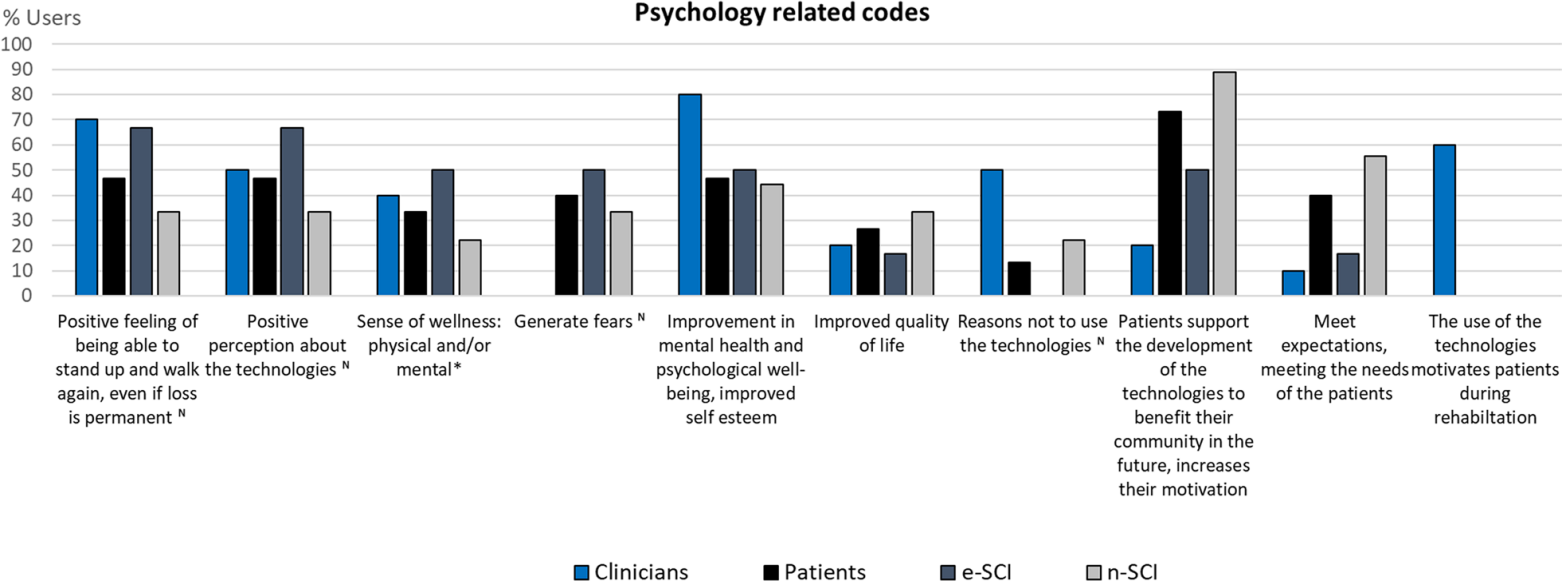
Codes related to Psychology (n = 10). The number of codes in this category demonstrates that the benefits expected from the use of WR for gait rehabilitation of SCI patients do not only concern physical benefits but also psychological benefits. In this category, patients overall showed high agreements in most codes. Almost half of both patients and clinicians have a positive perception about the technologies, about the feeling of being able to walk and stand up again even if their loss is permanent, and they expect improvements in the mental health and psychological well-being of patients thanks to the use of these devices. These are mostly expressed by e-SCI, showing the opportunities of the technologies in cases of people who have already used them. This should be a motivation to make these devices more accessible to their intended users. Additionally, especially n-SCI expressed they felt motivated to support the development of the technologies to benefit their community in the future, showing that patients are willing and eager to be included in the design and development processes. Four patients said the use of the technologies gave them a physical and/or psychological sense of wellness, but one n-SCI was more skeptical about this effect

**Fig. 8:**
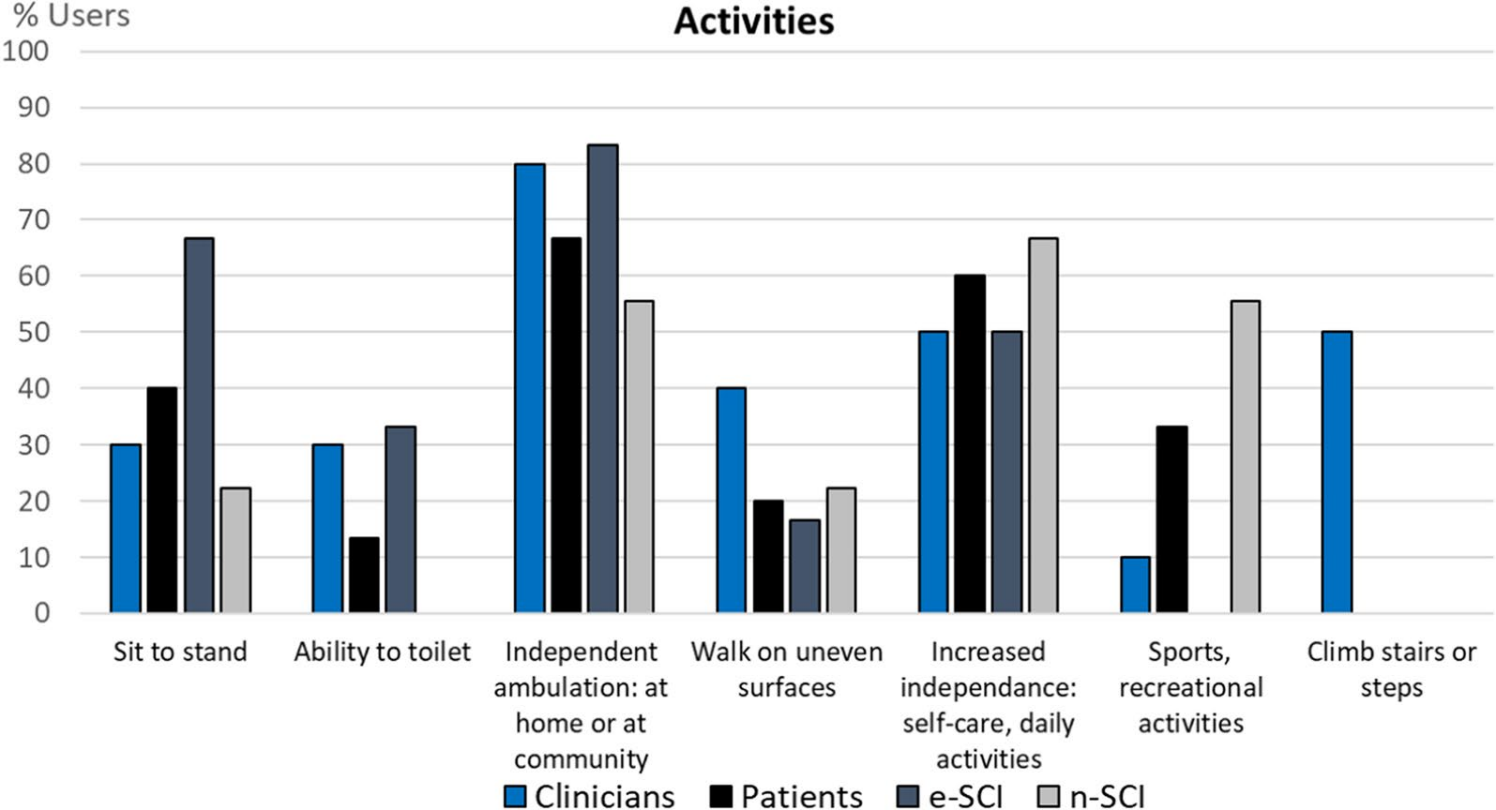
Codes of the category Activities (n = 7). In this category we grouped the activities that users would like to do with the technologies. All groups mostly highlighted that they expect the devices to allow them to walk independently and to allow them to do self-care and daily activities with independence. Interestingly, one e-SCI explicitly mentioned that the technology he tried was not ready to be used independently by him at home. Only n-SCI expect the device to enable them to do sports or recreational activities, perhaps because e-SCI have met the actual capabilities and limitations of currently available technologies whereas n-SCI have not. Lastly, clinicians expressed interest in the possibility to climb steps or stairs with the technology, since this is a rehabilitation task. No activities besides the ones found in the literature were expressed by the users interviewed

**Fig. 9:**
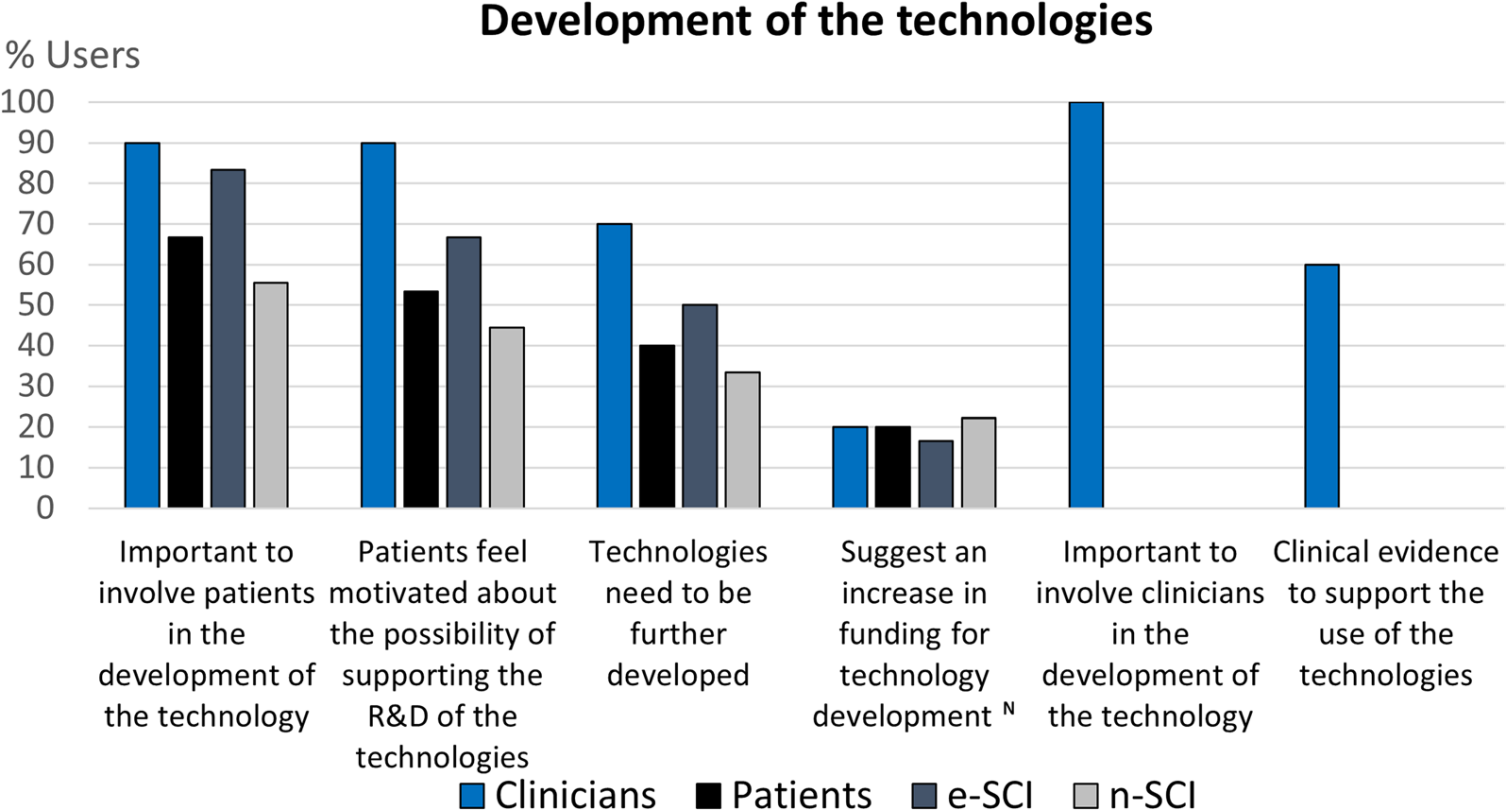
Codes related to the Development of the technologies (n = 6). In this category we grouped the codes related to the opinions of the users regarding the future development of the technologies. There is high agreement among users in the need to further develop the technologies but also in the urge to involve patients in the development of the technologies. Users say patients are the ones who know what they need and the requirements that they have as primary users of exoskeletons. At the same time, both groups of users recognize that patients feel motivated about the possibility of supporting the research and development of the technologies. Therefore, it is a win–win situation for developers and users to involve patients within the technology development cycle. The only new code that arose in this category was the call to increase the funding for the development of exoskeletons, and they expressed this is relation to governmental institutions. All clinicians said it is important to involve them in the developments as well, since they are the ones who know the rehabilitation needs of the patients, and the needs of the PT and PM&R within the real constraints of the health system. Most of them also talked about the lack of clinical evidence to support the use of the technologies, which is still matter of research, given the difficulties of performing randomized case-controlled trials in the field [23]

**Fig. 10:**
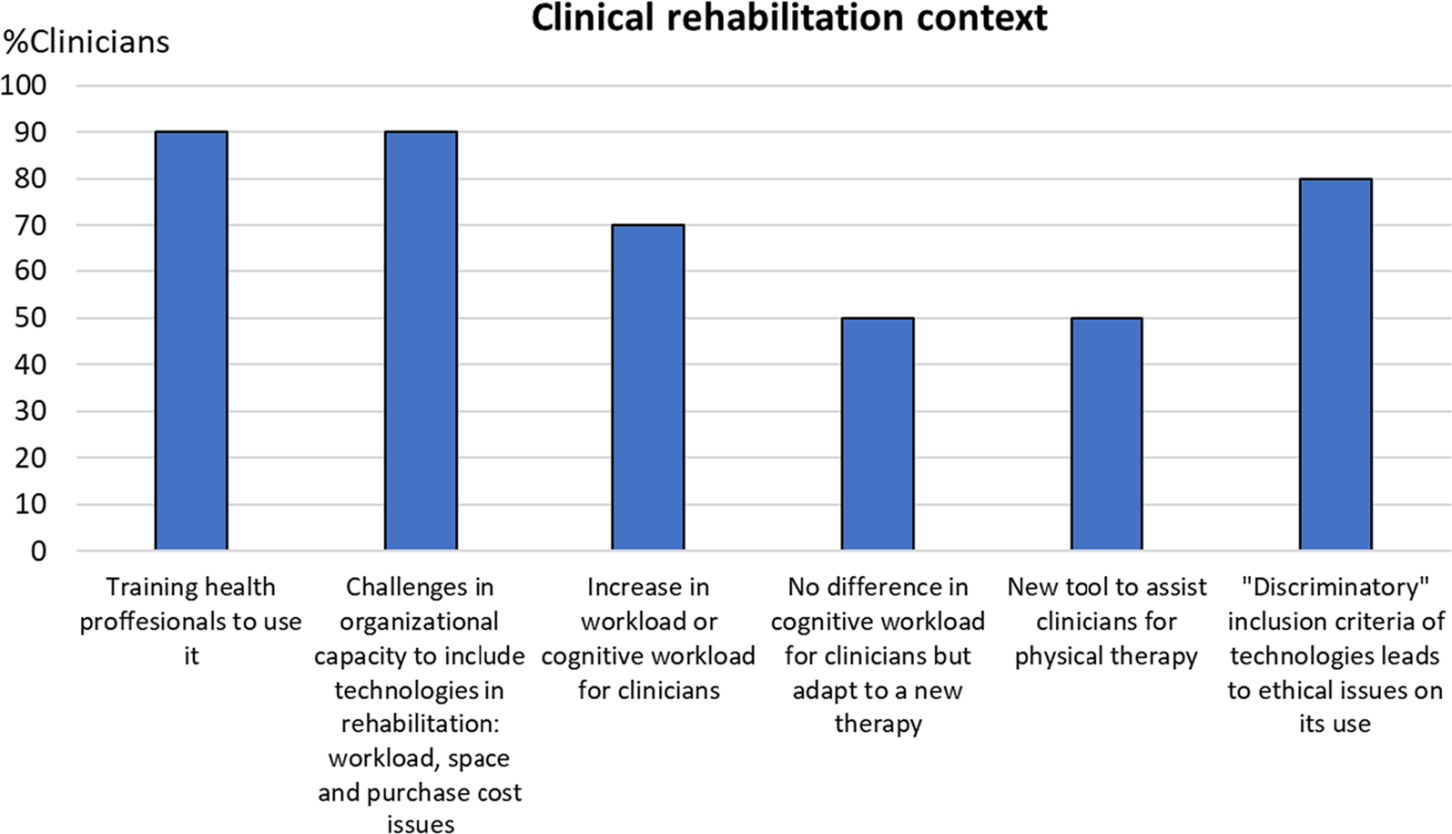
Codes related to the Clinical rehabilitation context (n = 6). This category has codes related to constraints of the clinical rehabilitation context that must be taken into account to ensure the feasibility of deploying exoskeletons within the health care facilities. Clinicians identify challenges related to the organizational capacity of hospitals to implement exoskeletons in rehabilitation such as space availability, high purchase cost and an increase in workload. Most clinicians (70.0%) consider that the use of exoskeletons will result in an increase in the physical and/or cognitive workload of the PT. However, 50% consider that the workload will not increase after they get adapted to exoskeletons as a new tool for therapy. Actually, the only new code in this category is precisely clinicians seeing exoskeletons as a new tool to assist them for physical therapy. Likewise, this is related to the importance of training health professionals to use the devices, a topic that 90% of them expressed. Most of the clinicians (80.0%) were concerned about the ethical issues regarding the selection of the patients that are prescribed to use the technologies, i.e. prescribing them only to patients that can benefit the most with the use of the exoskeletons but leaving out other patients that could still benefit from them, due to the limited devices available and their limited accessibility

**Fig. 11:**
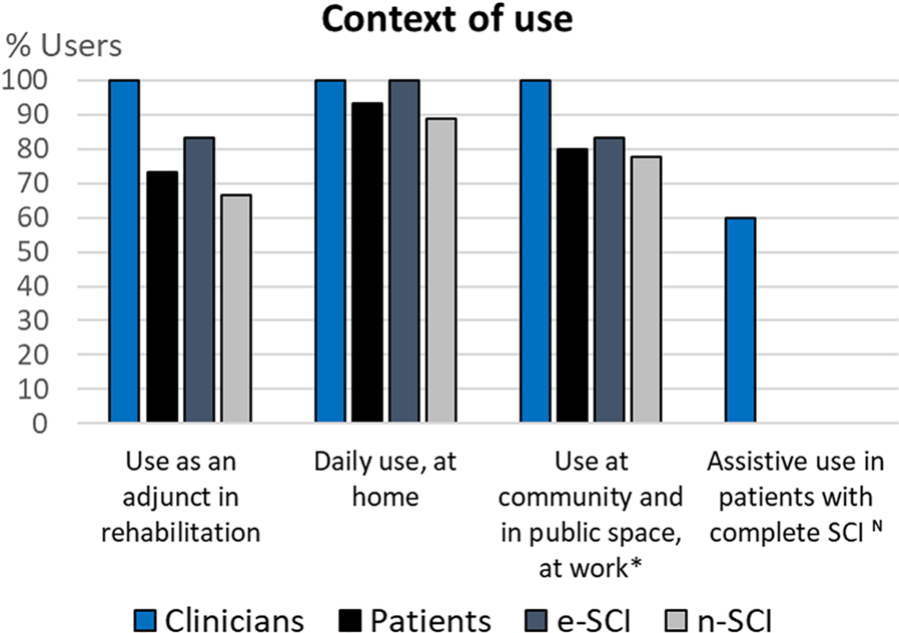
Codes related to the Context of use (n = 4). Most participants would like to use the technologies in rehabilitation settings, in their communities (e.g. public space or at work), and also in a daily basis at home. However, two patients said these technologies, in their current state, are not ready to be used at home. Similarly, one e-SCI said he would not “dare go outside wearing [an exoskeleton] and go for a beer two blocks away”. Clinicians (60%) also expect the technologies can assist complete SCI patients in their daily life. The latter is the only new code in this category

**Fig. 12:**
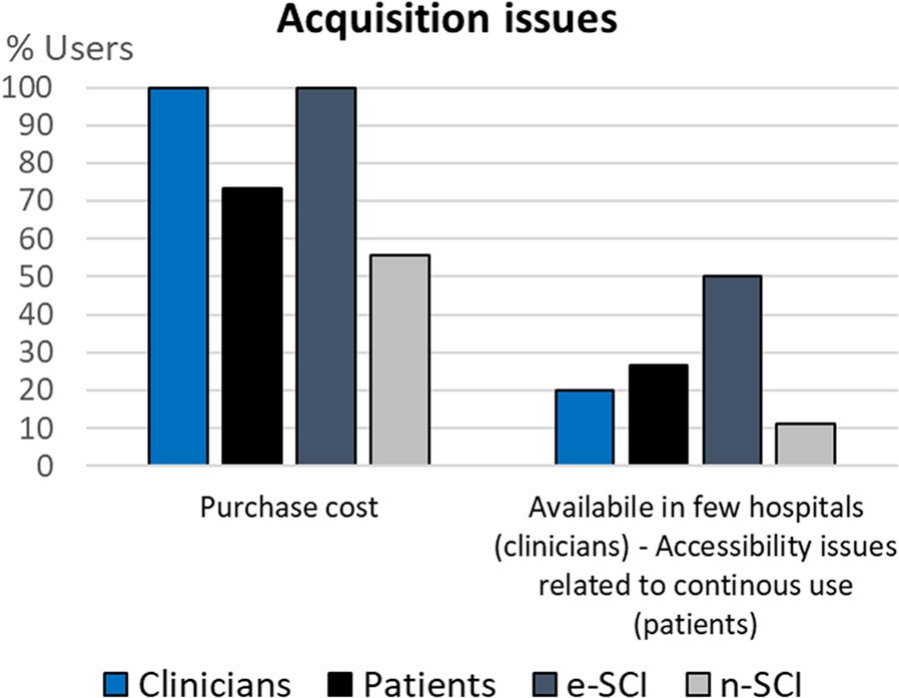
Codes related to Acquisition issues (n = 2). The main acquisition issue expressed by all experienced users (clinicians and e-SCI) and most n-SCI (55.6%) is the purchase cost, a well-known limitation of exoskeletons currently [23]. Some of the users, mostly e-SCI, also talked about the limited access to the technologies that are in hospitals or rehabilitation centers, both because there are few devices available and because they are busy most of the time, given that several users are assigned to each available device. No new codes emerged in this field

To visualize the codes expressed by at least 20% of clinicians or people with SCI, we use column charts showing the relative frequency of each group that referred to each code. Figures for each category are comprised within Figs. 4, 5, 6, 7, 8, 9, 10, 11, 12. In all graphs, the codes in the left side correspond to the ones expressed by more e-SCI than n-SCI, and the ones in the right to codes expressed by more n-SCI. The new codes that arose from our data are marked with the symbol (^N^). Some codes in the figures have a sign (*) at the end, representing that some users that talked about the same code but with a different perspective from the other users; these cases are detailed in the description of the corresponding figure.

In this category, two out of the four new codes that emerged were related to negative aspects such as 40% of the patients having fears about using exoskeletons or 50% of the clinicians and 22.20% of n-SCI having reasons to reject their use. Examples of the fears are: falling, damaging the device, “doing it wrong”, and hurting one-self and affecting body parts that are currently healthy. Patients’ reason not to use the technologies is that for their recovery and independence it is better to do all the activities they can without the exoskeleton, and they need to know their capabilities at the end of their sub-acute rehabilitation to assess if an exoskeleton is needed. For clinicians the reasons include: the need to adapt to a new technology that could be complex to use, lack of trust towards technologies due to the fear that they will replace physiotherapists in their workplace, having to lift and move heavy devices, and believing that traditional therapy is better than robot-assisted therapy.

## Discussion

Through qualitative research, we managed to determine a comprehensive set of requirements, perceptions, and expectations that people with spinal cord injury and the clinicians in charge of their rehabilitation have for the design of lower limb wearable exoskeletons for gait rehabilitation. To our knowledge, this is the first research comparing the expectations of people with SCI without experience in the use of exoskeletons versus the requirements of experienced users, and most importantly, the first study that aims to summarize a comprehensive list of criteria for the design of these technologies, encompassing the knowledge available in the literature and allowing new criteria to emerge from our own data. Both the criteria summarized in our study and the additional ones from literature (see Additional file 2: Annex 2), ought to guide developers of these technologies to make sure the efforts invested in the field lead to technologies that respond to the needs and expectations of their end users, comprising people with SCI as well as clinicians as secondary users. This will improve the availability and accessibility of the technologies, by designing devices that are feasible to be implemented in their intended contexts.

Our motivation to perform this comprehensive study is also a consequence of the increasing interest shown by developers and researchers in the field in understanding user perception and experience with lower-limb wearable exoskeletons, as seen by a steep increment in studies published in 2020 regarding the topic, performed with smaller samples and specific devices. The comprehensive and in-depth study presented here was possible thanks to the use of directed content analysis approach, a qualitative research methodology that allows to focus criteria elicitation from the user’s point of view.

Regarding the data collected in our study, the ratio between the average length of the interviews for each group and the number of codes that emerged (n-SCI: 34.0 min, 44 codes; e-SCI: 22.5 min, 38 codes; Clinicians: 21.2 min, 82 codes), shows even though less clinicians than patients were interviewed, they agreed on more codes in interviews that were almost 1/3^rd^ shorter. On the contrary, n-SCI are less accurate in expressing their requirements and have broader expectations about exoskeletons, as seen in the lowest agreement rate in the codes expressed (see Table 4) and the longest average interview length. This is a result of the limited knowledge and information that people out of the field have about these technologies. When interviewed, all n-SCI but one said they did not have any previous knowledge about exoskeletons before being admitted to the hospital. Of these, four patients did not have information about the technologies even after being in the hospital. When asked about what an exoskeleton for them was, they recalled movies or news as their only source of information to make a guess. The remaining four patients recognized the Lokomat as an exoskeleton for gait rehabilitation, available in the gym at the hospital. Only two n-SCI had seen a portable exoskeleton before.

In relation to intergroup agreement rates, the agreement percentage among both types of patients (55.77%) demonstrates the contrast between the expectations from n-SCI and the “down-to-earth” requirements from e-SCI, and therefore, the complementarity of their requirements. Indeed, including both types of patients in this study and people with injuries of different severity was aimed at seeking requirements representative of the heterogeneous SCI population. Similarly, the intergroup agreement percentage of 50.0% between patients and clinicians indicates that including both type of users in the design process of these technologies is a must, given that their requirements are complementary. As shown in our results, both patients and clinicians agree on the importance of involving both types of users in the design and development of lower limb wearable exoskeletons, and they are motivated and willing to participate in these processes [25, 58]. They are stakeholders of exoskeletons in different ways, thus, both must be taken into account to design technologies that are usable, respond to users’ needs and that are feasible to implement in their intended contexts. For most customers (i.e. individuals, hospitals, healthcare systems, or private rehabilitation institutions) the overall experience with a company, and not only the product itself (i.e. the exoskeleton), is fundamental to engage in business [62]. Actually, according to a report published in 2020 [62], 66% of customers expect companies to understand their unique needs and expectations, and healthcare sector is the one in which customers are concerned the most about being the center of the products and services. Understanding this important expectation will be fundamental for developers and companies in the field to develop technologies that are successful in reaching end users.

With respect to the agreement intragroup (see Fig. 3), clinicians show higher agreement percentages for more codes, with the whole group agreeing on six (6) codes (high purchase cost, use at community and in public space or at work, daily use at home, use as an adjunct in rehabilitation, easy control with different control options, important to involve clinicians in the development of the technologies). On the contrary, all the group of patients or n-SCI patients do not agree on any code, but all e-SCI patients interviewed did agree on two (2) codes (daily use at home, high purchase cost). In total, 18 codes expressed by clinicians were not mentioned by the patients (23.1% of all the codes). In general, for all the agreement quartiles over 25%, more codes were agreed on by clinicians than patients. In contrast, for all the agreement quartile below 25%, patients agreed on more codes than clinicians. This demonstrates that having experience with the technologies (clinicians and e-SCI) result in higher agreements and in having focused requirements within a group. In this regard, the experience with the technologies makes the e-SCI group similar to the clinicians in terms of expressing more requirements in shorter interviews and in higher agreement rates between both groups when compared to n-SCI. Therefore, involving clinicians and e-SCI in User-centered design (UCD) processes, even if they are few people, would be efficient and useful for developers because they give focused feedback representative of their respective requirements.

The new codes found in our data are one of the most important contributions of our paper. Considering the use of lower limb exoskeletons in rehabilitation settings, it is very important for PT that the devices have manageable weights and are easy to handle and move around, to not to increase the physical burden during therapy due to the manipulation of the device. All three groups agreed on the relevance of having devices that can be fit to different bodies and functionally adapted according to the patient needs, since these needs differ from user to user due to their specific impairment, and also evolve along therapy. Overall, there is a lack of scientific evidence to identify the specific population that can benefit from each technology depending on its features, as well as regarding the specific protocols that allow to optimize their use as rehabilitation devices [23].

Mostly clinicians and some e-SCI demand an improvement in the interaction of the device with the surroundings through sensors that help the device to automatically adapt to different scenarios, and an improvement in the interaction of the device with the person wearing it by closing the control loop through biofeedback and intention detection to move the exoskeleton. The latter is key to enhance neuroplasticity in robotic-assisted gait rehabilitation [1]. To these ends, both groups also referred to the advantages of combining lower limb exoskeletons with BCI or FES. Hybrid exoskeletons for gait rehabilitation are currently being explored due to the potential of adding the benefits of both types of technologies [63].

It is also very important to understand the fears that SCI people have regarding the use of these devices, because they ought to be addressed during the design of the technologies and when training the users, to allow them to trust the device and have a smooth interaction. Similarly, it is imperative to address clinicians’ concerns regarding the perceived threat of exoskeletons for walking rehabilitation through education. Exoskeletons for gait rehabilitation are not meant to replace physiotherapists, instead, they are meant to be a new tool to assist clinicians for physical therapy, exactly like the healthcare workers of our study expressed. Previous experiences with similar technologies, including for example the Lokomat, can show clinicians that these devices allow to provide intensive rehabilitation reducing the physical burden that PT have in traditional therapy, allowing them to (1) invest the time in more observation and evaluation of the progress of patients, (2) have more time available to design better therapy plans for patients and (3) have objective data regarding the patient movement and evolution, provided by the devices.

All types of users interviewed in this study suggest an increase in funding for lower limb exoskeletons, mostly because they consider these technologies are still under development. Nonetheless, currently there are six (6) devices CE marked, and there are at least four times more devices in different development stages [23], most of whom have been developed thanks to funding provided by public and private institutions. Then, why is it that at least 30 years of research, funding and dozens of developments aimed at the same goals have not provided at least a couple of devices that are not perceived by users as “still under development”? Perhaps the community of developers are not focusing enough in investing the funding available to develop technologies that are usable by their end users and perceived by them as close to be realistically available in their intended contexts. In fact, the limited accessibility and availability of lower limb exoskeletons for gait rehabilitation is a topic that arose in most of the interviews, demonstrating the limited devices that have successfully reached end users when compared to the demand for exoskeletons. To overcome this issue, researchers and developers ought to (1) improve the usability of their devices including usability evaluations of their devices following benchmarks [61, 64] and (2) implement UCD: people with SCI and clinicians expressed they are motivated to participate in the developments and that their involvement is fundamental, because, in their own words, “they are the ones who know what they need”. Even though they perceive the technologies still need years of development, almost half of the users interviewed have a positive perception about the technologies, especially e-SCI. They, together with clinicians, highlight the positive feeling of being able to stand up and walk again, even if loss is permanent. This demonstrates it is worthy to keep working on improving the devices, since experienced users see the potential they have, but developers must focus on meeting the actual needs of their end users and on addressing the constraints of their intended context of use.

## Implication

The complete set of criteria summarized in our study, encompassing both the knowledge available in the literature and the new criteria that emerged from our own data, will be useful to guide developers of WR for gait rehabilitation in the design, development and evaluation of their technologies, to make sure the efforts invested in the field lead to technologies that respond to the needs and expectations of their end users and are feasible to implement in their intended context of use. Additionally, we emphasize the need to implement User-centered design and usability evaluation in the field, in line with the findings of this study.

## Limitations

Qualitative research, due to its emphasis on in-depth understanding and context-specific insights, does not lend itself to statistical generalization of findings to the broader population [65]. Nevertheless, our sample has similar gender distribution to the incidence of SCI [66], conforms to the sample characteristics suggested in the literature to implement qualitative methodologies through content analysis of semi-structured interviews [39] and allowed to reach data saturation thanks to performing comprehensive semi-structured interviews regarding a specific topic. Moreover, it is larger than the samples of most other studies available in the literature implementing similar methodologies and related to the same topic. The only study with similar methodology but a larger sample [59] focuses in identifying only functional and design requirements for a soft exoskeleton, whereas our study aimed at identifying a more comprehensive set of requirements in different dimensions for the design of lower limb exoskeletons.

A potential limitation to the generalizability of the results is that the data collection was held in Spain, a country with a public health system. This could represent cultural differences in some of the expectations of users from other countries without a public health system, particularly those regarding the accessibility of the technologies and the constraints to effectively implement the technologies in clinical rehabilitation settings. Nevertheless, we consider user’s requirements in 6 out of the 9 categories presented in our study (physical results, technical characteristics, usability, psychology-related codes, activities, and development of the technologies) are most likely preserved within people with a spinal cord injury regardless their country of residence, since their demands depend more on the functional limitations caused by the injury and the intrinsic condition of being humans interacting with a robotic device than by cultural expectations.

Both the chronicity and injury severity differences between the subgroups of people with SCI are another limitation of this study, since they could have influenced the perspectives of the users interviewed. As indicated in the manuscript, the subjects were recruited through criterion and convenience sampling techniques, therefore, despite the research team sought for heterogeneity and managed to have both complete and incomplete SCI subjects in both patients’ subgroups, a more balanced sample was not feasible to be recruited for this study. Requirements of incomplete and complete SCI are indeed complementary instead of equivalent. The formers use WR for gait rehabilitation as tools to improve their ability to walk whereas the latter use them for gait rehabilitation mostly due to the physical and psychological benefits that these technologies represent, but not to improve their ability to walk. This is why it was imperative to gather insights in both subgroups and in the whole study from both types of subjects, to effectively summarize a comprehensive set of requirements from a heterogeneous sample of SCI subjects.

Regarding the chronicity of the injury, the marked difference is due to the type of centers where each subgroup was recruited. One is meant for subacute inpatient rehabilitation while the other is for chronic rehabilitation after discharge from the first institution. The research team aimed to recruit both subgroups only from HNP but there were no experienced subjects in the institution, given that no active or recent research was ongoing with WR devices for gait rehabilitation due to the pandemic. Therefore, an alternative center with people with SCI with experience in the use of these technologies had to be included in the study, and due to proximity, cultural, and convenience reasons, the FLM was the institution reached to recruit the e-SCI sample. In spite of this limitation, the authors consider that the chronicity of the injury could possibly affect mostly the expectations expressed between the acute and the beginning of the subacute phase of the injury, with respect to the chronic phase, because SCI subjects are reaching the plateau of their gait rehabilitation by the end of the subacute period of their injury (6 months approximately), where the enhanced period of neuroplasticity is over. Taking this into account, the average time since injury of the n-SCI is by the end of the subacute period, and therefore, no massive differences are estimated between the expectations of these subjects and the ones expressed by the e-SCI. The time undergone in intensive rehabilitation is also essential to understand the rehabilitation goals of each subject with the specific characteristics of their injury and their maximum expected recovery. Therefore, while undergoing the subacute period, people with an SCI learn to understand their injury and adapt their lives while seeking the maximum possible rehabilitation, where the WR for gait rehabilitation could help.

The research team was aware of these limitations regarding the differences in severity and chronicity of the injuries and decided to provide and analyze the overall requirements of SCI subjects as a whole and the subgroup statistics separately, to make both results available to developers and researchers in the field. In this way, this limitation does not affect the requirements summarized for the whole group of people with SCI.

## Conclusions

People with spinal cord injury and the clinicians in charge of their rehabilitation are stakeholders of exoskeletons with complimentary design requirements, thus, both must be involved in designing technologies that are usable, respond to users’ needs and that are feasible to implement in their intended contexts, because currently, there is limited accessibility and availability of lower limb exoskeletons for gait rehabilitation. This can be achieved through implementing User-centered design during the development of these technologies: users interviewed are motivated to participate in the developments and they agreed on the relevance of being involved. We provide a comprehensive set of requirements, perceptions, and expectations of these users for the design of lower limb wearable exoskeletons for gait rehabilitation that can serve as a starting point for developers.

### Supplementary Information

**Additional file 1:** Semi-structured interview guide.

**Additional file 2:** Full list of codes found in the study and in the literature survey.

## Data Availability

The required data to support the results of this study are available in the figures and text of the manuscript. Any reader with any further questions regarding the data and results, can contact the corresponding author.
