# Supplementary material for: A qualitative study to elicit user requirements for lower limb wearable exoskeletons for gait rehabilitation in spinal cord injury
Source: J Neuroeng Rehabil. 2023 Oct 17;20:138. doi: 10.1186/s12984-023-01264-y (PMC10583355; doi:10.1186/s12984-023-01264-y)
Supplement: Supplementary file 1 — Additional file 1: Semi-structured interview guide. [file 12984_2023_1264_MOESM1_ESM.pdf]

Annex 1. Questions included in the semi-structured interviews for each user group.

n-SCI (People with SCI without experience in the use of the technologies)

1. Why did you agree to participate in this process?
2. What do you know about exoskeletons (how they work, what the training process is like, possible benefits)? Where did you get this information on the subject?
3. What do you think of robotic technologies that provide walking assistance?
4. What expectations do you have about them?
5. How do you imagine these technologies? How would you like them to be?
6. What features would you find important about the technology? Think about physical aspects and related to its use.
7. In what contexts/situations would you like to use the devices?
8. What activities do you think it would allow you to do and with what level of independence?
9. Do you think that the use of technology will have any impact on a psychological level?
10. Do you expect to get any benefit in terms of your physical health with the use of these devices?
11. If you were to purchase the device, what factors would be important in making the decision to purchase it or not?
12. As a technology user, what do you think about being involved in the technology design and development process? What motivates you to get involved and what do you hope to gain from your participation?

e-SCI (People with SCI with experience in the use of the technologies)

1. Why did you agree to participate in this process?
2. What do you know about exoskeletons (how they work, what the training process is like, possible benefits)? Where did you get this information on the subject?
3. What do you think of robotic technologies that provide walking assistance?
4. What expectations do you have about them?
5. What has been your experience with exoskeletons, neuroprosthetics or hybrid robots?
6. How was the process of approaching and learning with the device? How did you learn to use it, what sensations did you have, are there specific experiences that you remember?
7. As a technology user, what do you think about being involved in the technology design and development process? What motivates you to get involved and what do you hope to gain from your participation?
8. What features would you find important about the technology? Think about physical aspects and related to its use.
9. In what contexts/situations would you like to use the devices?
10. What activities do you think it would allow you to do and with what level of independence?
11. Do you think that the use of technology will have any impact on a psychological level?
12. Do you expect to get any benefit in terms of your physical health with the use of these devices?

13. If you were to purchase the device, what factors would be important in making the decision to purchase it or not?

Clinicians related to gait rehabilitation of people with SCI and/or research with WR for gait rehabilitation in SCI and other neurological injuries.

1. Why did you agree to participate in this process?
2. What do you know about exoskeletons (how they work, what the training process is like, possible benefits)? Where did you get this information on the subject?
3. What do you think of robotic technologies that provide walking assistance?
4. What do you think about being involved in the technology design and development process? What motivates you to get involved and what do you hope to gain from your participation?

From your role in the patient's rehabilitation process:

5. What features and physical characteristics would you find important about the technology?
6. What activities would you want/need the device to allow the patient? With what level of independence?
7. Thinking about the use of the device, what factors are indispensable for your patient to be able to use it?
8. What processes or professional dynamics do you find important to be able to involve technology in your workplace and work activities?
9. In what contexts/situations would you recommend, and would you like to use the devices for your patient's rehabilitation?
10. Do you expect your patient to gain any physical health benefits from using the device?
11. Do you think that the use of technology will have any psychological impact on your patient?
